# Genetic heterogeneity and consanguinity lead to a “double hit”: Homozygous mutations of *MYO7A* and *PDE6B* in a patient with retinitis pigmentosa

**Published:** 2013-07-20

**Authors:** Nitza Goldenberg-Cohen, Eyal Banin, Yael Zalzstein, Ben Cohen, Ygal Rotenstreich, Leah Rizel, Lina Basel-Vanagaite, Tamar Ben-Yosef

**Affiliations:** 1 The Krieger Eye Research Laboratory, Felsenstein Medical Research Center, Schneider Children’s Medical Center, Beilinson Campus, Petah Tikva, Israel; 2 Sackler Faculty of Medicine, Tel Aviv University, Tel Aviv, Israel; 3 Department of Ophthalmology, Hadassah- Hebrew University Medical Center, Jerusalem, Israel; 4 Raphael Recanati Genetic Institute, Rabin Medical Center, Beilinson Hospital, Petah Tikva, Israel; 5 Department of Genetics, The Rappaport Faculty of Medicine and Research Institute, Technion – Israel Institute of Technology, Haifa, Israel; 6 Maurice and Gabriela Goldschleger Eye Research Institute, Sheba Medical Center, Tel-Hashomer, Israel; 7 Pediatric Genetics, Schneider Children’s Medical Center of Israel, Petah Tikva, Israel; 8 Felsenstein Medical Research Center, Rabin Medical Center, Petah Tikva, Israel

## Abstract

**Purpose:**

Retinitis pigmentosa (RP), the most genetically heterogeneous disorder in humans, actually represents a group of pigmentary retinopathies characterized by night blindness followed by visual-field loss. RP can appear as either syndromic or nonsyndromic. One of the most common forms of syndromic RP is Usher syndrome, characterized by the combination of RP, hearing loss, and vestibular dysfunction.

**Methods:**

The underlying cause of the appearance of syndromic and nonsyndromic RP in three siblings from a consanguineous Israeli Muslim Arab family was studied with whole-genome homozygosity mapping followed by whole exome sequencing.

**Results:**

The family was found to segregate novel mutations of two different genes: myosin VIIA (*MYO7A*), which causes type 1 Usher syndrome, and phosphodiesterase 6B, cyclic guanosine monophosphate-specific, rod, beta (*PDE6B*), which causes nonsyndromic RP. One affected child was homozygous for both mutations. Since the retinal phenotype seen in this patient results from overlapping pathologies, one might expect to find severe retinal degeneration. Indeed, he was diagnosed with RP based on an abnormal electroretinogram (ERG) at a young age (9 months). However, this early diagnosis may be biased, as two of his older siblings had already been diagnosed, leading to increased awareness. At the age of 32 months, he had relatively good vision with normal visual fields. Further testing of visual function and structure at different ages in the three siblings is needed to determine whether the two RP-causing genes mutated in this youngest sibling confer increased disease severity.

**Conclusions:**

This report further supports the genetic heterogeneity of RP, and demonstrates how consanguinity could increase intrafamilial clustering of multiple hereditary diseases. Moreover, this report provides a unique opportunity to study the clinical implications of the coexistence of pathogenic mutations in two RP-causative genes in a human patient.

## Introduction

Retinitis pigmentosa (RP) is the most common form of hereditary retinal degeneration (HRD) with a worldwide prevalence of 1 in 4,000. RP is the most genetically heterogeneous group of disorders in humans. RP reflects a heterogeneous group of pigmentary retinopathies characterized by night blindness followed by visual-field loss, often resulting in severe visual impairment. In most patients, the disease is limited to the eye (nonsyndromic), and is usually inherited as autosomal recessive (AR), autosomal dominant, or X-linked [1]. More than 60 genes have been implicated in nonsyndromic RP (RetNet). More than 30 forms of syndromic RP have also been described; the most common is Usher Syndrome (USH), with a worldwide prevalence of 3–10 per 100,000 (reviewed in [2]). USH is inherited in an AR mode, and is characterized by the combination of RP and bilateral sensorineural hearing loss (SNHL), with or without vestibular dysfunction. The most severe form is Usher type 1 (USH1), characterized by profound prelingual SNHL, vestibular areflexia, and prepubertal onset of RP. To date, six USH1 causative genes have been identified [3,4].

Due to the marked genetic heterogeneity of RP, mutations in more than one RP causative gene may appear within the same family. Such cases have been previously reported in extended families [5]. Here, we report a rare case of a nuclear consanguineous family in which three of four siblings are affected with RP due to mutations of myosin VIIA (*MYO7A*, underlying USH1) [6] and/or phosphodiesterase 6B, cyclic guanosine monophosphate-specific, rod, beta (*PDE6B*, underlying nonsyndromic RP) [7].

## Methods

### Subjects

Family members were recruited following approval by the National Helsinki Committee for Genetic Research in Humans (Jerusalem) and by the local institutional review board at Rambam Medical Center in Haifa. Written informed consent was obtained from all participants or their parents. Patients underwent a detailed ophthalmic examination, including best visual correction, color vision, confrontational visual field, pupil response to light, funduscopy and fundus photographs, electroretinography (ERG), and optical coherence tomography (OCT).

### DNA analysis

Venous blood samples were obtained using K3EDTA vacuette tubes (Greiner Bio-One, Kremsmunster, Austria), and genomic DNA was extracted using high salt solution according to a standard protocol [8]. Genome-wide homozygosity mapping was performed using the HumanCytoSNP-12v2.1 BeadChip (220 K; Illumina, San Diego, CA). Homozygous regions were calculated using HomozygosityMapper [9]. Whole exome sequencing (WES) was performed at Otogenetics Corporation (Norcross, GA) using a Roche NimbleGen (Madison, WI) V2 (44.1 Mbp) paired-end sample preparation kit and Illumina HiSeq2000. A total of 5.1 gigabases (Gb) of paired-end 100-nucleotide sequence reads were obtained for each sample, 90% of which were mapped to gene regions following alignment to the human genome reference sequence (hg19) using the DNAnexus software package. Average exome coverage was 45 reads per nucleotide (coverage range 4–10,000). Variants were called and annotated according to the dbSNP database (build 129) using the DNAnexus software package.

For mutation confirmation and segregation analysis, specific primers were used to PCR amplify *MY07A* exon 20 (5ʹ-TCC TGA TCC CAA ACC CAC-3ʹ and 5ʹ-TGC CAT GGC AGC AAG GCT-3ʹ) and *PDE6B* exon 11 (5ʹ-ACG GTC ATT TGT CTC CAG AT-3ʹ and 5ʹ-AGT CAG GCC CAC TAA ACA TC-3ʹ). Mutation screening was performed with direct sequencing with the Big Dye terminator cycle sequencing kit on an ABI 3130×l Genetic Analyzer (PE Applied Biosystems, Foster City, CA).

## Results

**Figure 1:**
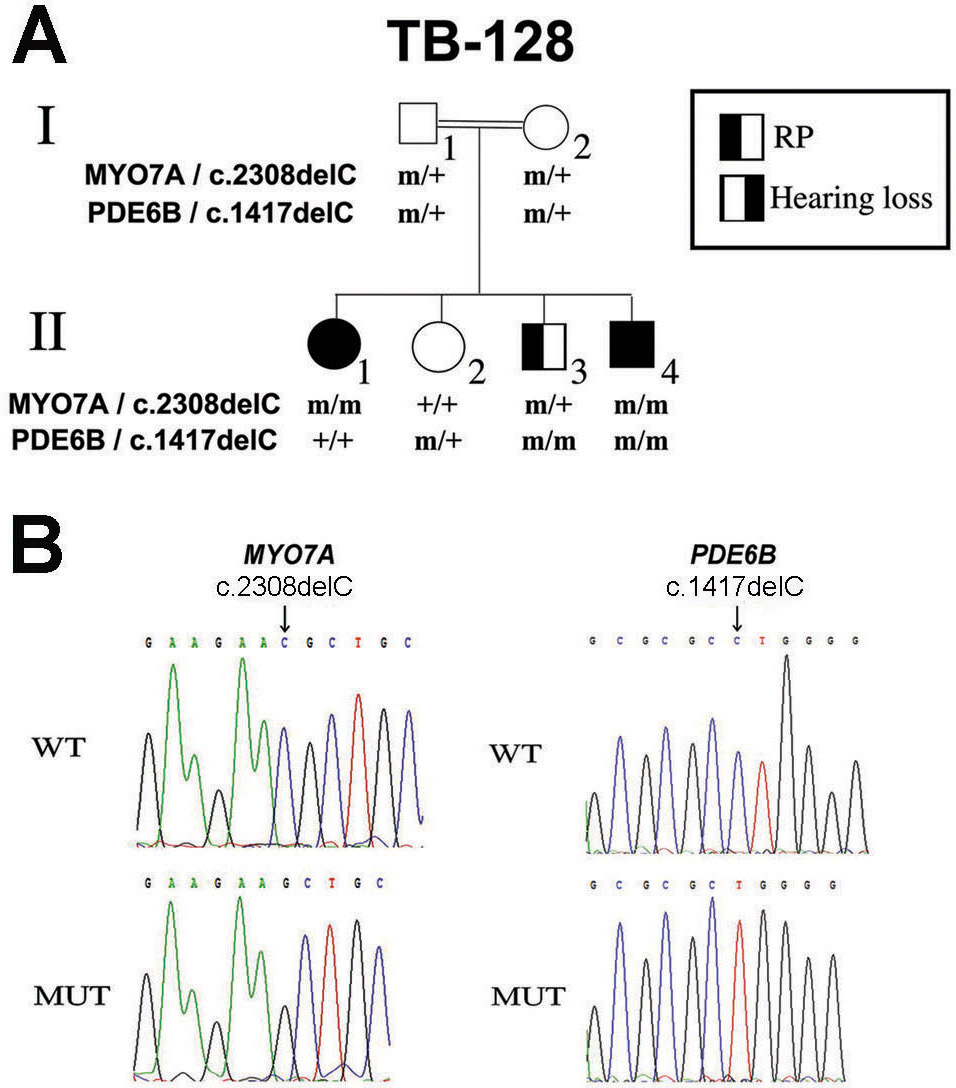
Pedigree and mutation analysis. **A**: Shown is a consanguineous Israeli Muslim Arab family segregating autosomal recessive sensorineural hearing loss (SNHL) and retinitis pigmentosa (RP). Double lines indicate a consanguineous union. Filled symbols represent affected individuals, whereas clear symbols represent unaffected individuals. The genotypes of each family member at the myosin VIIA (*MYO7A*) and the phosphodiesterase 6B, cyclic guanosine monophosphate-specific, rod, beta (*PDE6B*) genes are indicated. m, mutant allele; +, wt allele. **B**: Nucleotide sequence traces in non-carrier individuals (WT) and in affected individuals (MUT) homozygous for the c.2308delC mutation of *MYO7A* or the c.1417delC mutation of *PDE6B*.

Family TB128 is an Israeli Muslim Arab family. The parents are first cousins. Three of their four offspring (Figure 1A, individuals II:1, II:3, and II:4) were diagnosed with RP at the ages of 10 years, 7 years, and 9 months, respectively. Individual II:3 has normal hearing and motor development, while individuals II:1 and II:4 have bilateral profound SNHL, which was detected in infancy. They received cochlear implants at the ages of 3 years and 9 months, respectively. Both manifested a delay in motor development and walked independently only at 20–24 months of age. The combination of RP, congenital profound SNHL, and vestibular dysfunction is consistent with a diagnosis of USH1 [3]. A fourth sibling has normal hearing and vision.

Initially, the gene encoding connexin 26 (the most common cause of hereditary HL worldwide) [10] of individual II:1was sequenced, and her DNA was screened for known USH mutations using the Asper microarray system [11]. Both tests were negative.

When affected siblings are the outcome of a consanguineous marriage, the assumption is that their disease is due to homozygosity for the same recessive mutant allele. However, it is hard to explain the existence of USH1 and nonsyndromic RP in three siblings by a single mutation. To date, the only USH gene associated with nonsyndromic RP is *USH2A*, underlying USH2 (in which HL is not profound and vestibular function is usually normal) [3,12]. In USH3, there may be intrafamilial variability regarding the age of onset of HL; however, it is usually post-lingual and progressive [13,14]. Mutations of USH1 genes have not been associated with nonsyndromic RP, and partial penetrance has not been reported [3]. One exception is a recently identified mutation of *USH1C*, which causes RP associated with late-onset and relatively mild HL. These patients might be initially diagnosed with nonsyndromic RP [15]. Based on these facts, our working hypothesis was that family TB128 might segregate recessive mutations of two different genes, independently causing RP and HL, and mimicking the USH1 phenotype. Similar cases have been previously reported [16].

To identify these genes, we performed genome-wide homozygosity mapping using DNA samples from the three RP-affected individuals. One of the homozygous intervals shared among these three individuals included a known RP-causative gene (nuclear receptor subfamily 2, group E, member 3; *NR2E3*); however, screening of all coding exons was negative for mutations. We then looked for homozygous intervals shared between the two deaf individuals only. We found a 44 Mb interval on chromosome 11 that included the *MYO7A* gene, underlying USH1B [6]. We next performed WES on DNA samples from individuals II:1 (HL and RP) and II:3 (RP only). A homozygous deletion of a C nucleotide at position 2308 of *MYO7A* cDNA (GenBank accession number NM_000260), located in exon 20 (c.2308delC), was identified in individual II:1 (Figure 1B). This novel mutation is expected to lead to a frameshift and premature termination of translation after insertion of four irrelevant amino acids, starting at position 769 of the myosin VIIA protein (p.N769*fs*X4). Individual II:3 was heterozygous for this mutation, and no other *MYO7A* mutation was detected.

The homozygosity data for individual II:3 showed multiple homozygous intervals, which included three known retinal degeneration-causative genes: IQ motif containing B1 (*IQCB1*), rhodopsin (*RHO*), and *PDE6B*. WES data revealed a deletion of a C nucleotide at position 1417 of *PDE6B* cDNA (GenBank accession number NM_000283), located in exon 11 (c.1417delC; Figure 1B). This novel mutation is expected to lead to a frameshift and premature termination of translation after insertion of 16 irrelevant amino acids, starting at position 473 of the PDE6B protein (p.L473*fs*X16). This mutation was found homozygously in individual II:3 and was not detected in individual II:1. Both mutations were confirmed with direct Sanger sequencing. Segregation analysis in the family revealed that parents were heterozygotes for both mutations, while the unaffected sister was heterozygote for the *PDE6B* mutation only. Interestingly, the third affected brother (individual II:4) was homozygous for both mutations (Figure 1A).

**Figure 2:**
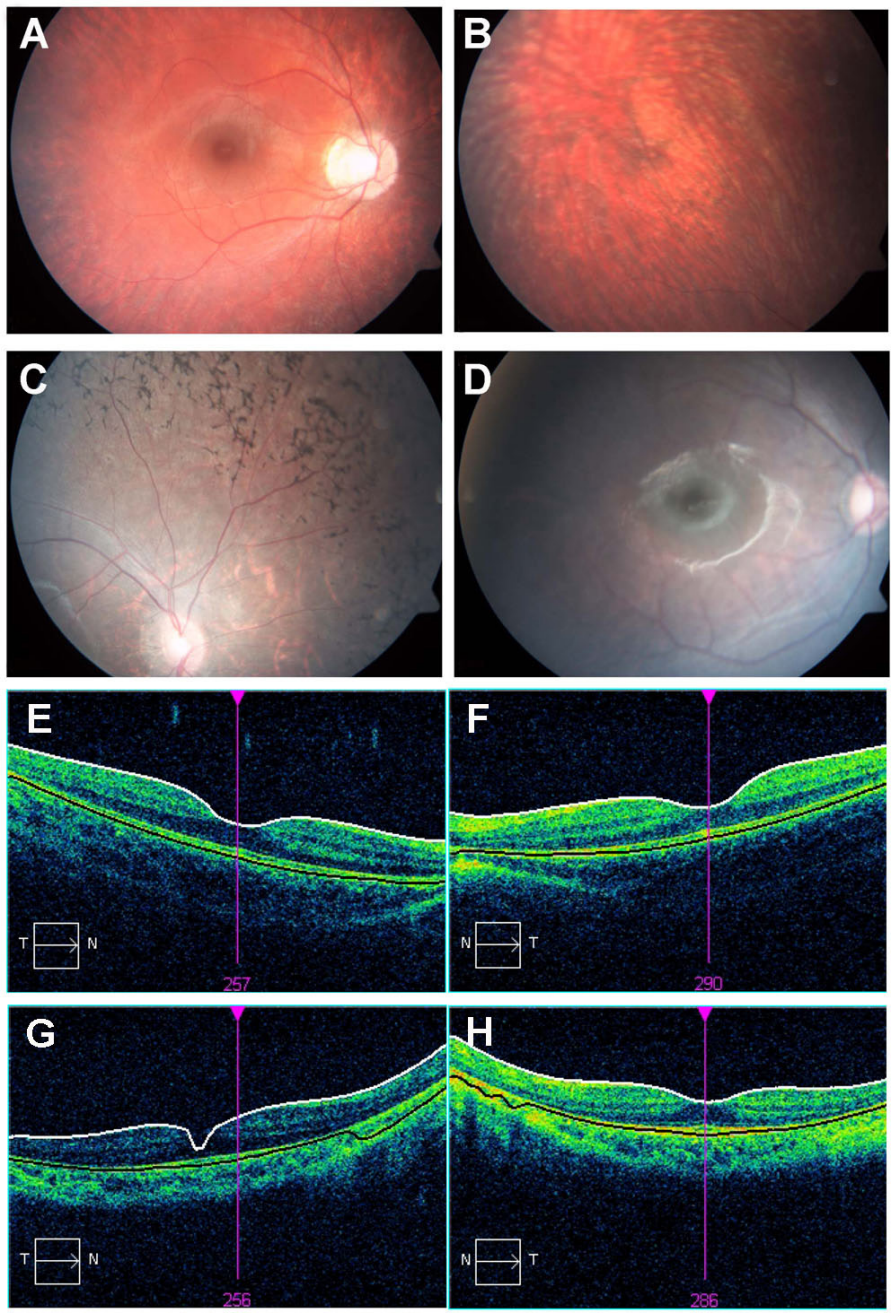
Fundus photographs and optical coherence tomography (OCT) of affected individuals from family TB128. **A**, **B**: Fundus photographs of individual II:1 at the age of 15 years demonstrate normal optic disc and blood vessels with peripheral temporal degenerative changes and atrophy of the retina. **C**: Fundus photograph of individual II:3 at the age of 13 years demonstrates waxy pallor of the tilted optic discs with severe attenuation of the retinal blood vessels, and bone spicule-like pigmentation in the entire retinal periphery. **D**: Fundus photograph of individual II:4 at the age of 32 months demonstrates oval optic disc with temporal peripapillary atrophy, normal color, mild attenuation of retinal blood vessels, and no pigmentary changes in the retina at this stage. **E**, **F**: Optical coherence tomography (OCT) of the right and left eyes (RE and LE, respectively) of individual II:1 demonstrates normal retinal thickness. Macular thickness: RE 274 μm, LE 260 μm. Mean optic nerve fiber layer thickness: RE 101 μm, LE 104 μm. **G**, **H**: OCT of RE and LE, respectively, of individual II:3 demonstrates normal retinal thickness. Macular thickness: RE 262 μm, LE 246 μm. Mean optic nerve fiber layer thickness: RE 105 μm, LE 97 μm.

Ophthalmologic evaluation was performed in all family members. Clinical findings are summarized in Table 1. Both parents had normal vision with no pathological findings. Individual II:3 (*PDE6B* mutation homozygote) was first diagnosed with RP at the age of 7 years. At this age, the cone and rod ERG responses were recordable, but reduced, with the rods more severely affected (Table 1). At the age of 13 years, he had constricted visual fields, waxy pallor of the optic discs, severe attenuation of retinal blood vessels, and severe bone spicule-like pigmentation in the entire periphery (Figure 2C). His older sister (II:1, *MYO7A* homozygote) had a milder retinal phenotype. At the age of 12 years, the cone and rod ERG responses were recordable, but reduced, with the rods more severely affected (Table 1). At the age of 15 years, her visual fields were normal, and the degenerative changes observed in her retina were relatively mild with mild attenuation of the arteries (Figure 2A,B). OCT imaging in II:1 and II:3 showed normal macular thickness (Figure 2E–H). The youngest sibling (individual II:4) is homozygote for *PDE6B* and *MYO7A* mutations. His retinal dysfunction was diagnosed as early as 9 months of age, based on severely reduced ERG responses measured using a short protocol (without full dark adaptation; Table 1). At the age of 32 months, his confrontational visual field appeared normal, and he had mild attenuation of retinal blood vessels, with no overt pigmentary changes (Figure 2D). Full-field ERG rod responses recorded at 3 years of age following 40 min of dark adaptation were non-detectable, and the mixed cone-rod response was severely attenuated. Cone responses were similarly severely reduced and delayed (Table 1). Notably, his brother (II:3), who is homozygous for the *PDE6B* mutation only, had better conserved cone ERG responses at the older age of 7 years. His rod responses were similarly non-detectable, and mixed cone-rod responses also severely reduced (Table 1). The oldest sister (II:1), who is homozygous for the *MYO7A* mutation only, had lower mixed cone-rod and cone ERG responses at the age of 12 years (Table 1).

**Table 1:** Clinical data of affected individuals from family TB128

Patient ID	Genotype	Vision											Hearing
		Age at Diagnosis of Vision Impairment	Visual Acuity (best corrected)	Visual Field^║^	Color Vision†	Funduscopic Findings	FFERG ¶						
							Age at FFERG Testing	LA Cone Flicker		DA Mixed Response Amplitude		DA Rod Response	
								Amp(μV)	IT (msec)	a wave (μV)	b wave (μV)	Amp (μV)	
II-1	*MYO7A* c.2308delC hom	10 y	R 6/10 L 6/7.5 (15y)	BE Full (15y)	Ish, BE normal (15y)	Cells in vitreous; Retina: peripheral temporal atrophy, degenerative changes in periphery, with mild attenuation of the blood vessels (15y)	12 y	9	42	24	22	10	Congenital, bilateral profound SNHL
II-3	*MYO7A* c.2308delC het; *PDE6B* c.1417delC hom	7 y	BE 6/8.5 (13y)	BE peripheral temporal loss (mainly of the lower temporal VF) (13y)	Ish, BE normal (13y)	Optic discs: tilted waxy pallor; severe attenuation of retinal blood vessels; marked bone spicule-type pigmentation in the entire periphery (13y)	7 y	31	37	26	55	NR	Normal
II-4	*MYO7A* c.2308delC hom; *PDE6B* c.1417delC hom	9 m	BE 6/15 (32m)	BE Full (32m)	Abnormal‡ Did not follow color tracks (32m)	Optic discs are oval with temporal peripapillary atrophy; mild attenuation of retinal blood vessels, no pigmentary changes (32m)	9 m	19	40	ND	ND	ND	Congenital, bilateral profound SNHL
							3 y	20	43	26	78	NR	

Hom, homozygote; het, heterozygote; y, years; m, months; R, Right eye; L, Left eye; BE, both eyes; SNHL, sensorineural hearing loss ^║^Visual Field: confrontation †Color Vision: Ish=Ishihara PseudoIsochromatic plates color test ‡Did not follow color tracks, possibly due to misunderstanding of test instructions ¶ FFERG=Full field electroretinography, including the following details: Light-adapted (LA) Cone flicker amplitude (Amp, normal 60–144 μV) and implicit time (IT, normal 27–33 msec). Dark-adapted (DA) mixed cone-rod a and b-wave amplitudes (normal a-wave 90–350 μV, normal b-wave 380–630 μV); Dark-adapted rod response b-wave amplitude (normal range 200–500 μV). NR=Non Recordable; ND=Not done; In patients II-1 and II-4 electroretinographic responses were largely symmetric, so presented as average of the two eyes. In patient II-3 only the RE was tested.

## Discussion

HRD is a strikingly heterogeneous condition, associated with mutations in more than 100 genes and loci (RetNet) [1].The contribution of each one of these genes to the overall prevalence of retinal degeneration is relatively small. However, the total number of pathogenic mutations in all HRD-causative genes is relatively elevated in the general population, and it is estimated that approximately 1 in 4–5 individuals may be a carrier of null mutations responsible for HRD. Due to the highly heterogenous nature of HRD, such an elevated frequency of unaffected carriers has a limited influence on the likelihood of generating affected offspring, since the chance of two unrelated parents carrying each a heterozygous mutation in the same gene remains low. However, the risk for consanguineous couples of generating a child with HRD is particularly high, compared to other genetic conditions [17]. Here, we present a rare case of three siblings with the same retinal phenotype (RP) and three different genotypes (*MYO7A* deficiency, *PDE6B* deficiency, or both).

In the retina, most myosin VIIA is localized in retinal pigmented epithelial (RPE) cells, where the protein is involved in organelle motility and in light-dependent translocation of the visual cycle enzyme RPE65. A small amount of myosin VIIA is also associated with the connecting cilium of photoreceptor cells, where it may be involved in maintaining a diffusion barrier (reviewed in [18]).

*PDE6B* encodes the β subunit of rod photoreceptor cyclic guanosine monophosphate (cGMP)-phosphodiesterase (PDE), a key enzyme of the visual phototransduction cascade. The holoenzyme is a heterotetrameric complex, consisting of α, β, and two identical γ subunits. Activation of cGMP-PDE leads to reduced intracellular concentration of cGMP, thus closing cGMP-gated cation channels located on the rod plasma membrane and initiating a neural response to light [19]. Malfunctioning of cGMP-PDE results in elevated cGMP concentrations, which may lead to an excessive energy load on rod photoreceptors, resulting in degeneration [20]. Indeed, mutations in the genes encoding each of the subunits α, β, and γ, cause arRP [7,21,22].

The mutations we identified in *MYO7A* and *PDE6B* are truncating and consequently expected to be null. The retinal phenotypes observed in individuals II:1 and II:3 are consistent with those previously reported in other patients with mutations in *MYO7A* and *PDE6B*, respectively [6,7]. The retinal phenotype seen in patient II:4, who is homozygous for mutations in both genes, results from overlapping pathologies that include a combination of cellular defects affecting RPE function, visual cycle, and photoreceptor cell structure and survival. An intriguing question is whether this “double hit” translates into more severe manifestation of the retinal disease. The young age at which this child was diagnosed with RP (9 months) could suggest increased severity, but it may simply reflect increased awareness of the parents and treating physicians following the presence of disease in two of his older siblings and the profound deafness that was apparent early on. ERG testing performed in this child at the age of 3 years, as detailed above, may suggest a somewhat more severe degenerative process at least as compared with the brother manifesting RP due to the homozygous *PDE6B* mutation, who had a better preserved cone function at the older age of 7 years. However, further testing of visual function and structure over time in the three siblings is needed to determine whether the fact that two RP-causing genes are mutated in this youngest sibling confers increased disease severity. Interestingly, a patient homozygous for mutations of *PDE6B* and G protein-coupled receptor 98 (*GPR98*), the gene underlying USH2C, has been previously reported. Her retinal phenotype was indeed more severe than that of her affected relatives who were homozygous for mutations in either of these two genes [5]. The current report reinforces the genetic heterogeneity of RP, and demonstrates how consanguinity could increase intrafamilial clustering of multiple hereditary diseases. Moreover, this report provides a unique opportunity to study the clinical implications of the coexistence of null mutations in two RP-causative genes in a human patient.
